# Distinct Populations of Neurons Activated by Heroin and Cocaine in the Striatum as Assessed by catFISH

**DOI:** 10.1523/ENEURO.0394-19.2019

**Published:** 2020-02-03

**Authors:** Philip Vassilev, Riccardo Avvisati, Eisuke Koya, Aldo Badiani

**Affiliations:** 1 Sussex Addiction Research and Intervention Centre (SARIC), School of Psychology, University of Sussex, Brighton BN1 9RH, United Kingdom; 2 Department of Physiology and Pharmacology, Sapienza University of Rome, 00185 Rome, Italy

**Keywords:** addiction, neuroplasticity, nucleus accumbens, opioid, psychostimulant, striatal complex

## Abstract

Despite the still prevailing notion of a shared substrate of action for all addictive drugs, there is evidence suggesting that opioid and psychostimulant drugs differ substantially in terms of their neurobiological and behavioral effects. These differences may reflect separate neural circuits engaged by the two drugs. Here we used the catFISH (cellular compartment analysis of temporal activity by fluorescence *in situ* hybridization) technique to investigate the degree of overlap between neurons engaged by heroin versus cocaine in adult male Sprague Dawley rats. The catFISH technique is a within-subject procedure that takes advantage of the different transcriptional time course of the immediate-early genes *homer 1a* and *arc* to determine to what extent two stimuli separated by an interval of 25 min engage the same neuronal population. We found that throughout the striatal complex the neuronal populations activated by noncontingent intravenous injections of cocaine (800 μg/kg) and heroin (100 and 200 μg/kg), administered at an interval of 25 min from each other, overlapped to a much lesser extent than in the case of two injections of cocaine (800 μg/kg), also 25 min apart. The greatest reduction in overlap between populations activated by cocaine and heroin was in the dorsomedial and dorsolateral striatum (∼30% and ∼22%, respectively, of the overlap observed for the sequence cocaine–cocaine). Our results point toward a significant separation between neuronal populations activated by heroin and cocaine in the striatal complex. We propose that our findings are a proof of concept that these two drugs are encoded differently in a brain area believed to be a common neurobiological substrate to drug abuse.

## Abstract (precis)

Despite the still prevailing notion of a shared substrate of action for all addictive drugs, there is evidence suggesting that opioid and psychostimulant drugs differ substantially in terms of their neurobiological and behavioral effects. These differences may reflect separate neural circuits engaged by the two drugs.

## Significance Statement

Despite significant advances in the substance use disorders field, effective prevention and treatment strategies are scarce and still under active development. Here we add to growing evidence indicating major differences in the neurobiological effects of opioid versus psychostimulant drugs, which is at odds with the still prevailing notion of a shared substrate of action for all addictive drugs. This suggests that, to be effective, the development of prevention and treatment strategies should not look for a “silver bullet” solution to all drug addictions. Instead, they should be tailored to the specific drug preference of pathologic users.

## Introduction

Virtually all current theories of drug abuse posit that the addictive properties of drugs depend on common neurobiological processes, including hyper-reactivity of motivational systems (Wolf, 2010; Berridge and Robinson, 2016), impaired impulse control (Jentsch and Taylor, 1999), and aberrant learning (Everitt and Robbins, 2005). Regardless of the core process on which each theory focuses, the biological substrate of said processes involves the mesotelencephalic dopamine (DA) system projecting from ventral tegmental area (VTA) and substantia nigra to the striatal complex, including caudate and nucleus accumbens (NAcc), and to the prefrontal cortex (PFC). Indeed, it is commonly assumed that all substances of abuse increase dopamine levels in the terminal regions of the dopaminergic system (Di Chiara and Imperato, 1988; Robinson and Berridge, 1993; Wise, 1996; Nestler, 2001, 2004; Hyman et al., 2006; Koob and Volkow, 2010; Berridge, 2012; Covey et al., 2014; Keiflin and Janak, 2015; Volkow and Morales, 2015; Berridge and Robinson, 2016; Keramati et al., 2017; Volkow et al., 2017) albeit via different mechanisms of action. Psychostimulant drugs, such as cocaine and amphetamines, produce dopamine overflow by binding the dopamine transporter (for review, see Kuczenski et al., 1982; Johanson and Fischman, 1989). Opioid agonists, such as heroin and morphine, are thought to increase dopamine concentrations indirectly by binding μ-opioid receptors located on inhibitory interneurons in the VTA; hence, disinhibiting dopaminergic neurons (Gysling and Wang, 1983; Matthews and German, 1984; Johnson and North, 1992). Yet, the magnitude of drug-induced dopamine overflow differs enormously from one drug to another, even within the same pharmacological class. For example, some opioids produce dramatic increases in dopamine, whereas others have very little effect (Gottås et al., 2014; Vander Weele et al., 2014). Furthermore, electrophysiological experiments have shown that neurons in the striatum respond in a very different manner to heroin versus cocaine self-administration (Chang et al., 1998; Wei et al., 2018), suggesting that the effects of the two drugs are encoded differently in this brain area.

**Figure 1.**
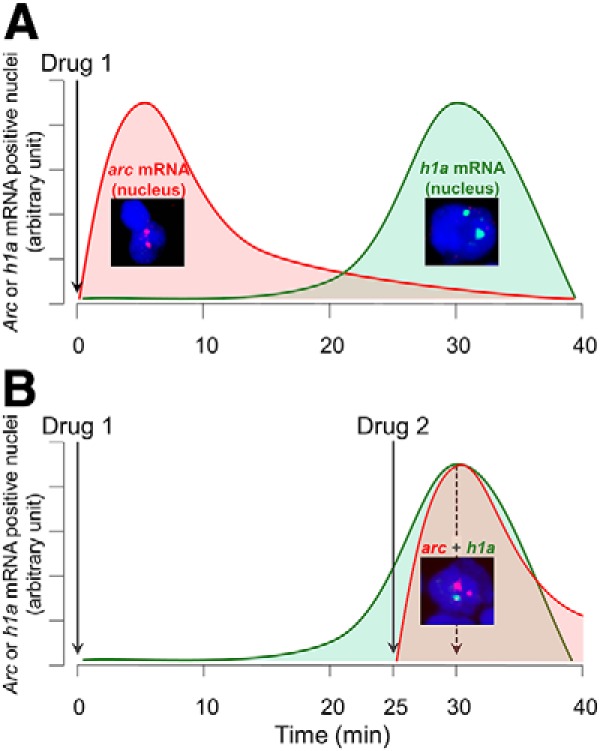
The catFISH paradigm. Working hypothesis based on the review by Guzowski et al. (2005): the expression of mRNA encoding for *h1a* and *arc* should be detectable at different time points after drug administration. ***A***, *arc* mRNA expression in the nucleus should peak at ∼5 min after drug administration, whereas *h1a* mRNA should peak at ∼30 min. ***B***, Overlap in the expression of Drug 1-induced *h1a* mRNA and Drug 2-induced *arc* mRNA should be observed at time 30 min (25 min after Drug 1 and 5 min after Drug 2).

The aim of the two experiments reported here was to further explore this hypothesis using the catFISH (cellular compartment analysis of temporal activity by fluorescence *in situ* hybridization) technique, which is a within-subject technique that takes advantage of the different transcriptional time course of the immediate-early genes (IEGs) *homer 1a* (*h1a*) and *arc* to detect the activation of partly distinct neuronal populations in response to two temporally distinct stimuli (Fig. 1; Guzowski et al., 1999; Vazdarjanova et al., 2002; Vazdarjanova and Guzowski, 2004). To date, a few studies have looked at the effects of cocaine on *arc* ( Caffino et al., 2011) or *homer 1a* expression (Unal et al., 2009), whereas there is no information on the effects of heroin administration on the expression of these two IEGs. As in the case of the IEG c-*fos*, which is known to be transcribed across the striatum in response to heroin and cocaine administration (Harlan and Garcia, 1998; Paolone et al., 2007; Celentano et al., 2009), both *arc* and *homer 1a* are activated by the transcription factor CREB; that is, they are transcribed following the activation of the ERK/MAPK pathway, elevated cAMP activity, or calcium influx to the cell (Impey et al., 1998; Sato et al., 2001; Kawashima et al., 2014). Considering these shared mechanisms of expression, we expected that *arc* and *homer 1a* would be suitable markers of neuronal activity produced by drug administration. We predicted that intravenous injections of heroin and cocaine will produce a rapid and transient IEG transcription in the striatum. Indeed, we found that intravenous administration of low doses (i.e., those typically used in self-administration experiments) of heroin and cocaine produce temporally distinct increases in the expression of *h1a* and/or *arc* suggesting that both drugs induce neuronal activity across the striatum. In a second experiment, we used the catFISH technique to establish to what extent this activity occurs in overlapping versus drug-specific neuronal populations. Based on electrophysiological evidence suggesting distinct neuronal activity produced by heroin versus cocaine (Chang et al., 1998), we predicted that the administration of heroin following cocaine would activate nonoverlapping neuronal populations across the striatum.

## Materials and Methods

### Subjects

A total of 66 male Sprague Dawley rats [*n* = 37 in experiment (Exp) 1 and *n* = 29 in Exp 2] from ENVIGO were tested at a weight of 300–375 g. The rats were housed and tested in a temperature- and humidity-controlled room (21 ± 1°C; 50%) with a reversed 12 h light/dark cycle (lights on at 7:00 P.M.). The rats were housed in groups of three or four until surgery and individually thereafter. Food and water were provided *ad libitum* except during testing sessions. All regulated procedures were conducted in accordance with the UK 1986 Animal Scientific Procedures Act (ASPA) and received approval from the relevant Animal Welfare and Ethics Review Board. After their arrival in the animal facilities, the rats were given a period of at least 7 d before undergoing experimental procedures.

### Drugs

Anesthesia was induced with 110 mg/kg ketamine (Anesketin, Dechra) and 2 mg/kg xylazine (Rompun, Bayer HealthCare). Cocaine and heroin hydrochloride (Johnson Matthey-MacFarlan Smith) were dissolved in sterile saline and infused intravenously at the doses specified in the next paragraphs. Each infusion consisted of a volume of 40 μl of the appropriate drug solution delivered over 4 s. Saline-treated rats received equivalent volumes of saline.

### Intravenous catheter surgery

The surgical procedures were similar to those recently described by Avvisati et al. (2019). Briefly, after anesthesia, an 11 cm silicone catheter (0.37 mm inner diameter, 0.94 mm outer diameter), sheathed at 3.4 cm from its proximal end by a silicone bead, was implanted in the right jugular vein, externalized at the nape of the neck, and attached to a cannula secured to the top of the skull with dental cement. Following surgery, rats were allowed to recover for at least 7 d. Catheter patency was maintained by flushing the catheters daily with 0.1 ml saline.

### Catheter patency test

At the appropriate time (see next sections), the rats were killed via an intravenous infusion of pentobarbital (120 mg/kg in 200 μl of saline) through the catheter. This also served as a catheter patency test: the rats that did not become ataxic and die within 5 s would be excluded from the data analysis. All catheters were found to be patent.

### Drug administration procedures

#### Experiment 1

After recovery, the rats received, while briefly restrained, an intravenous infusion of either 400 μg/kg cocaine (*n* = 18) or 50 μg/kg heroin (*n* = 19) in their home cage. These doses were selected based on the findings of previous self-administration experiments (Caprioli et al., 2007b, 2008). The rats received the lethal pentobarbital injection and were then decapitated at different time points after the cocaine or heroin infusion, as follows: 0 min (*n* = 3 for both the cocaine and heroin groups), 8 min (*n* = 3 for both the cocaine and heroin groups), 16 min (*n* = 4 for both the cocaine and heroin groups), 25 min (*n* = 4 for both the cocaine and heroin groups), and 35 min (*n* = 4 and *n* = 5 for the cocaine and heroin groups, respectively).

#### Experiment 2

After recovery, the rats were moved to testing chambers used for self-administration experiments (PRS Italia; Avvisati et al., 2019). To reduce the potentially confounding effects of environmental novelty on drug-induced IEG expression (Uslaner et al., 2001; Paolone et al., 2007), we let the rats habituate to these chambers for 18 h before tethering them to the infusion lines. Food and water were available *ad libitum* during this habituation period and were removed immediately before tethering. The use of self-administration chambers allowed us to deliver drug infusions remotely via a computer-controlled infusion pump. The infusion pumps were programmed to start automatically, in the absence of the experimenter, 1 h after tethering. In this way, we avoided the confounding effects usually associated with signaled drug administration (Crombag et al., 1996) and/or handling. All rats received two intravenous infusions, 25 min apart, of the following: saline–saline (*n* = 4), cocaine 800 μg/kg–saline (*n* = 6), cocaine 800 μg/kg–cocaine 800 μg/kg (*n* = 6), cocaine 800 μg/kg–heroin 100 μg/kg (*n* = 6), or cocaine 800 μg/kg–heroin 200 μg/kg (*n* = 7). To administer two separate injections through the same catheter, the infusion lines were backfilled with the appropriate drug solutions, separated by a tiny air bubble, just before tethering of the rats. The rationale for using higher doses of cocaine and heroin in Exp 2 was to boost the magnitude of IEG expression. These doses were still within the range of those used in self-administration experiments (Zito et al., 1985; Dai et al., 1989; Roberts et al., 1989; Pettit and Justice, 1991; Shaham and Stewart, 1994; Wise et al., 1995; Mantsch et al., 2001; Wee et al., 2007; Mandt et al., 2012).

Five minutes after the second infusion, the rats were given 120 mg/kg pentobarbital, i.v., and, after decapitation, their brains were snap frozen in isopentane at −50°C.

### Brain slicing

**Figure 2.**
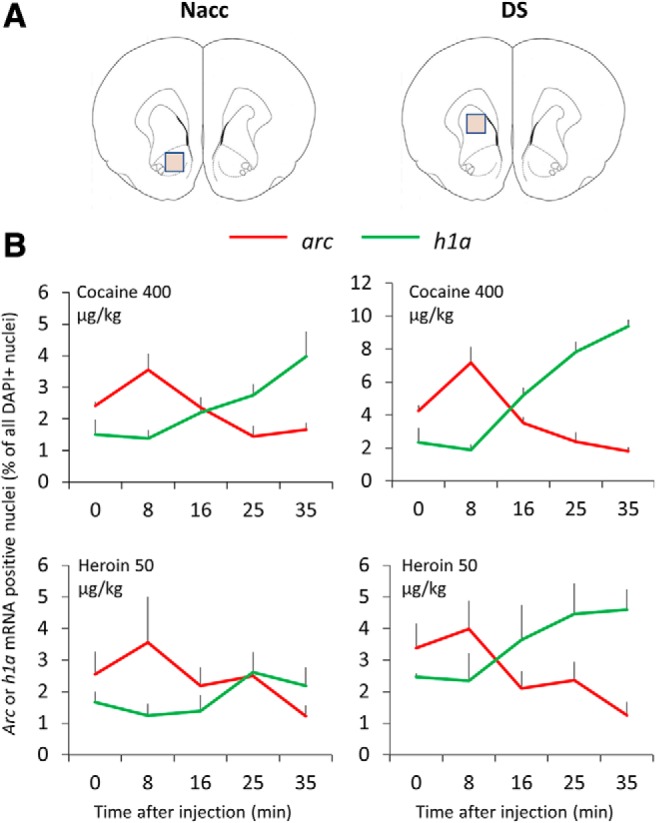
Effect of single drug injections on IEG expression. Time course of *arc* mRNA and *h1a* mRNA expression in experiment 1. ***A***, Regions of interest (plate from Paxinos and Watson, 1986). ***B***, Average number of *arc-* or *h1a*^+^ cell nuclei as a function of brain area and administered drug (expressed as a percentage of all DAPI^+^ nuclei). The brains were excised at different time points after drug administration, as follows: 0 min (*n* = 3 for both the cocaine and heroin groups), 8 min (*n* = 3 for both the cocaine and heroin groups), 16 min (*n* = 4 for both the cocaine and heroin groups), 25 min (*n* = 4 for both the cocaine and heroin groups), and 35 min (*n* = 4 and *n* = 5 for the cocaine and heroin groups, respectively).

**Figure 3.**
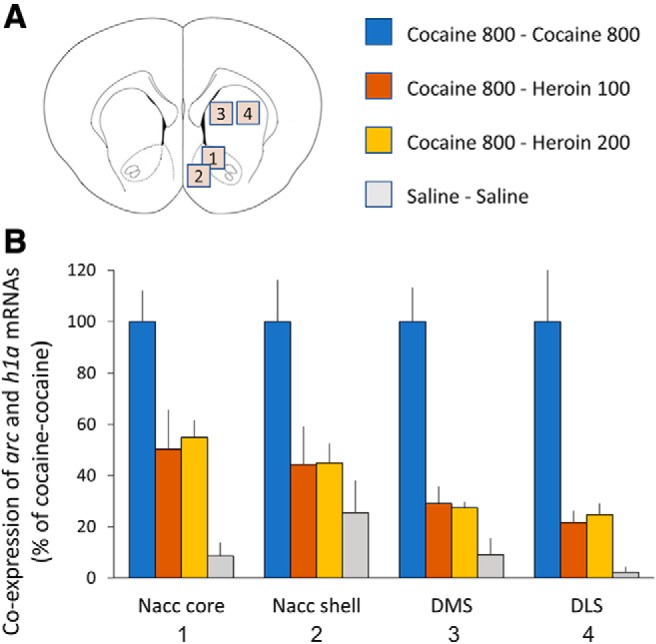
Overlap in the neuronal populations engaged by heroin and cocaine. Coexpression of *arc* and *h1a* mRNAs in experiment 2. ***A***, Regions of interest (plate from Paxinos and Watson, 1986). ***B***, Overlap expressed as the percentage of overlap in the cocaine–cocaine condition as a function of brain area and drugs administered, 25 min apart, in Exp 2: saline–saline (*n* = 4), cocaine (800 μg/kg)–saline (*n* = 6), cocaine (800 μg/kg)–cocaine (800 μg/kg; *n* = 6), cocaine (800 μg/kg)–heroin 100 μg/kg (*n* = 6), and cocaine (800 μg/kg)–heroin 200 μg/kg (*n* = 7).

The brains were excised and snap frozen in 400 ml of isopentane cooled to −50°C and later sectioned on a cryostat at 16 or 20 μm thickness. In Exp 1, sectioning started from the tip of the olfactory bulbs and brain sections were removed until the Sylvian fissure no longer reached the midline (+3.70 mm from bregma). At this point, either 100 or 80 sections were removed (when sectioning at 16 and 20 μm, respectively) to reach +2.00 mm from bregma at which point the sections contained anterior dorsal striatum (DS) and NAcc core (Fig. 2*A*). Two coronal sections per rat (16 or 20 μm thick) were obtained at this point. An identical procedure was used in Exp 2 to collect two coronal sections containing NAcc core and shell, dorsomedial striatum (DMS), and dorsolateral striatum (DLS) at +1.70 mm from bregma (Fig. 3*A*).

### *In situ* hybridization

Immediately after cutting, the brain tissue sections were mounted on Superfrost Plus slides. On the first day of staining, the slides were incubated in 10% neutral buffered formalin (catalog #HT501128-4L, Sigma-Aldrich) for 20 min at 4°C, followed by 2× 1 min washes in 1× PBS, and then serial dehydration in ascending concentrations of ethanol (5 min incubation in 50%, 70%, and 2× 100%). Following this, the tissue was stored in 100% ethanol overnight. On day 2, the tissue was air dried and then incubated with protease for 20 min, followed by 2× 1 min washes in distilled H_2_O. Protease, probe, and amplifier solutions were supplied by Advanced Cell Diagnostics (ACDbio) as part of a commercially available RNAscope Kit (ACDbio). *Arc* and *h1a* hybridization probes (catalog #317071-C2 and #433261, respectively, ACDbio) were hybridized to fresh frozen brain coronal sections sliced on a Leica CM1900 cryostat. The signal was amplified with an RNAscope Multiplex Fluorescent Reagent Kit (catalog #320850, ACDbio). The *arc* probe targeted the region spanning 1519–2621 bp of the *arc* gene mRNA (accession No. NM_019361.1). The *h1a* probe targeted the 3′ untranslated region of the *h1a* gene mRNA, spanning 5001–5625 bp (accession #U92079.1).

The *arc* and *h1a* probes were applied (50 μl/section), and the sections were incubated for 2 h at 40°C in a humidity-controlled oven. After incubation with the probes, the signal was amplified at four separate stages with 15, 30, 15, and 30 min of incubation in between (respectively) at 40°C in the hybridization oven. The probe and amplifier solutions were applied to the sections with the help of a hydrophobic pen barrier. There were 2× 2 min washes in wash buffer after each incubation (including after probe hybridization). Finally, sections were coverslipped and counterstained with DAPI mounting medium (catalog #H-1500, Vector Laboratories) and left at 4°C overnight.

### Image acquisition and analysis

Fluorescent signal was detected using a Zeiss Axioskop 2 Plus epifluorescent microscope, and images were acquired using AxioVision software (Zeiss).

Grayscale images were taken from both hemispheres of two adjacent sections for each rat at 20× magnification. This yielded four images per brain area for each rat. Final counts of DAPI-, *arc*-, and *h1a*^+^ nuclei were averaged from these four images. The resulting images represented a region of interest of 700 × 550 μm. These images were analyzed using the RIO Montpelier extension of the ImageJ software (Baecker and Travo, 2006). Grayscale images were analyzed separately for each channel—DAPI, Alexa Fluor 488 (*h1a*) and Cy3 (*arc*)—as follows. First, each DAPI image was analyzed by applying a Gaussian blur filter (sigma = 2), then a “rolling ball” background subtraction algorithm (ball radius = 20), followed by the application of the default automatic global thresholding algorithm. This yielded a binary image, which was then used to count objects selected on the basis of their size and circularity using the “analyze particles” function of ImageJ. The size criterion was set to 0.0045–0.045 square inches, and the circularity—to 0.7–1.00. This analysis resulted in a binary mask image containing only objects fulfilling the aforementioned criteria.

The images from the Alexa Fluor 488 and Cy3 channels were first adjusted for brightness so that the most visible signal was that coming from nuclear staining for *arc* and *h1a*. This was defined as any signal representing one or two bright dots close to each other, as opposed to the cytoplasmic signal, which is less bright and more diffused (Guzowski et al., 1999). A global threshold was then applied to the images (default algorithm), and the “analyze particles” function was used again to select only objects of 4–90 square pixels and to create a binary image mask showing only the defined particles.

A Windows 10 Dell OptiPlex 7060 desktop computer ran a MATLAB script to overlay the three binary mask images and count instances where objects defined as DAPI nuclei coincided with objects defined as *arc* mRNA, *h1a* mRNA, or both. The MATLAB code will be made available on request. Thus, IEG expression was measured by counting DAPI^+^ cell nuclei also positive for *h1a*, *arc*, or both.

### Statistical analyses

The data from Exp 1 were analyzed by two-way mixed ANOVAs, with time and IEG as fixed factors. The number of IEG^+^ cell nuclei (as a percentage of all DAPI-stained nuclei) was the dependent variable. The data from Exp 2 were analyzed using a two-way ANOVA, with brain area and treatment group as fixed factors. The outcome variable was overlap (expressed as a percentage of the cocaine–cocaine group). All analyses were conducted in SPSS 25 software (IBM). An α value of ≤0.05 was used for determining statistically significant effects.

## Results

### Experiment 1 (time course of *Arc* and *h1a* expression following intravenous drug administration)

Figure 2*B* shows the amount of *arc* and *h1a* ^+^ nuclei in the NAcc core and DMS expressed as a percentage of all DAPI^+^ nuclei and as a function of time elapsed since intravenous injections of cocaine and heroin. Table 1 shows the same data before conversion to a percentage.

**Table 1:** Mean (SE) number of *arc*- and *h1a*-stained cell nuclei as a function of brain area and drug administered in Exp 1

	NAcc				DS			
	Cocaine (400 μg/kg)		Heroin (50 μg/kg)		Cocaine (400 μg/kg)		Heroin (50 μg/kg)	
	Arc	H1a	Arc	H1a	Arc	H1a	Arc	H1a
0 min	19.50 (1.52)	12.50 (4.44)	18.42 (6.86)	11.33 (1.8)	30.10 (1.97)	16.42 (5.85)	21.17 (6.59)	14.83 (1.02)
8 min	25.50 (5.36)	9.75 (2.38)	26.92 (13.66)	8.83 (2.71)	44.58 (4.43)	11.83 (1.91)	26.92 (8.21)	14.58 (5.27)
16 min	16.31 (3.35)	14.81 (1.22)	13.88 (3.63)	8.63 (2.94)	21.81 (3.08)	32.19 (3.73)	12.31 (2.86)	20.88 (5.59)
25 min	9.94 (2.78)	17.81 (0.82)	18.19 (3.95)	18.69 (4.29)	15.00 (3.89)	48.88 (5.99)	16.44 (3.76)	31.75 (7.3)
35 min	11.00 (1.67)	26.25 (5.13)	8.00 (2.22)	14.6 (4.26)	11.25 (1.44)	58.50 (1.52)	7.50 (2.2)	28.35 (4.58)

The brains were excised at different time points after drug administration: 0 min (*n* = 3 for both the cocaine and heroin groups), 8 min (*n* = 3 for both the cocaine and heroin groups), 16 min (*n* = 4 for both the cocaine and heroin groups), 25 min (*n* = 4 for both the cocaine and heroin groups), and 35 min (*n* = 4 and *n* = 5 for the cocaine and heroin groups, respectively)

#### *Arc* and *h1a* expression in the NAcc core

Cocaine administration increased both *arc* and *h1a* mRNA levels in the NAcc core, but at different time points. A two-way mixed ANOVA showed nonsignificant main effects of IEG (*F*_(1,13)_ = 0.08, *p* = 0.782, *η^2^* = 0.006) and time (*F*_(4,13)_ = 1.62, *p* = 0.227, *η^2^* = 0.333), but a significant time × IEG interaction (*F*_(4,13)_ = 7.93, *p* = 0.002, *η^2^* = 0.977).

Heroin produced a similar pattern of mRNA expression, but the effect did not reach significance: a two-way mixed ANOVA showed nonsignificant main effects of IEG (*F*_(1,14)_ = 2.32, *p* = 0.150, *η^2^* = 0.142) and time (*F*_(4,14)_ = 0.72, *p* = 0.596, *η^2^* = 0.17), and a nonsignificant time × IEG interaction (*F*_(4,14)_ = 2.15, *p* = 0.129, *η^2^* = 0.38).

#### *Arc* and *h1a* expression in the DMS

As in the NAcc core, cocaine treatment increased IEG levels in a time-dependent manner. A two-way mixed ANOVA showed significant main effects of IEG (*F*_(1,13)_ = 18.93, *p* = 0.001, *η^2^* = 0.593) and time (*F*_(4,13)_ = 5.36, *p* = 0.009, *η^2^* = 0.623), and a significant time × IEG interaction (*F*_(4,13)_ = 44.58, *p* < 0.001, *η^2^* = 0.932).

Heroin produced a similar effect. A two-way mixed ANOVA showed nonsignificant main effects of IEG (*F*_(1,14)_ = 3.17, *p* = 0.097, *η^2^* = 0.185) and time (*F*_(4,14)_ = 0.22, *p* = 0.924, *η^2^* = 0.059), but a significant time × IEG interaction (*F*_(4,14)_ = 3.58, *p* = 0.033, *η^2^* = 0.506).

### Experiment 2 (overlap in neuronal populations activated by cocaine and heroin)

**Figure 4.**
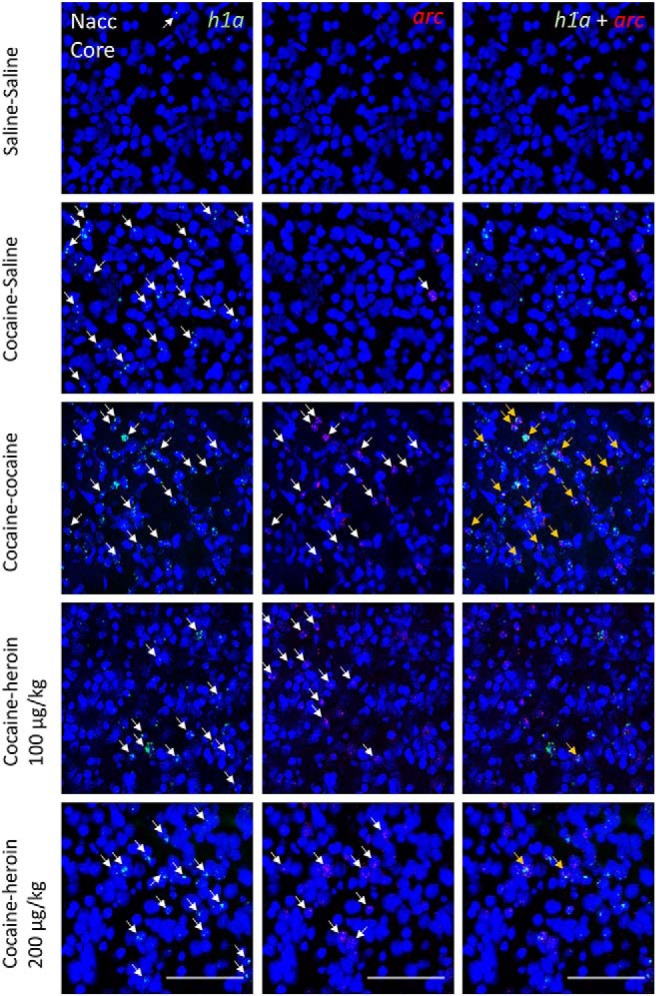
Representative microscope images taken from the NAcc core. DAPI-stained cell nuclei (blue) coexpress only *h1a* (green), only *arc* (red), or both. The columns show green and red channels separately and then merged. Taken from Nacc core. Scale bars, 0.1 mm. Arrows point to mRNA^+^ nuclei.

Table 2 shows the average number of *arc*-only, *h1a*-only, and double-stained cell nuclei as a function of brain area and drugs administered in experiment 2. Figure 4-Figure 7 show representative images from all brain areas analyzed using catFISH.

**Table 2.** Mean (SE) number of *h1a*-only, *arc*-only, and double-stained cell nuclei as a function of brain area and drugs administered, 25 min apart, in Exp 2: saline–saline (*n* = 4), and cocaine (800 μg/kg)–saline (*n* = 6), cocaine (800 μg/kg)–cocaine (800 μg/kg; *n* = 6), cocaine (800 μg/kg)–heroin 100 μg/kg (*n* = 6), and cocaine (800 μg/kg)–heroin 200 μg/kg (*n* = 7)

		NAcc core	NAcc shell	DMS	DLS
First saline Second saline	*h1a*	4.94 (0.66)	4.44 (1.61)	5.98 (2.18)	8.5 (2.54)
	*arc*	2.38 (0.94)	2.48 (0.75)	1.98 (0.95)	3.75 (1.59)
Double		0.13 (0.07)	0.38 (0.16)	0.13 (0.07)	0.13 (0.13)
First cocaine (800 μg/kg) Second saline	*h1a*	21.5 (4.44)	8.67 (2.36)	51.1 (7.99)	65.1 (7.83)
	*arc*	4.25 (0.77)	3.13 (0.68)	5.58 (1.77)	4.33 (0.95)
Double		1.46 (0.25)	1.67 (0.35)	3.63 (0.43)	4.46 (1.49)
First cocaine (800 μg/kg) Second cocaine (800 μg/kg)	*h1a*	20.54 (5.45)	11.00 (2.87)	42.17 (8.65)	49.71 (7.5)
	*arc*	8.08 (0.59)	5.13 (0.67)	12.54 (1.99)	15.33 (3.89)
Double		5.46 (1.04)	3.33 (0.77)	14.17 (3.17)	21.33 (5.19)
First cocaine (800 μg/kg) Second heroin (100 μg/kg)	*h1a*	20.33 (3.72)	8.04 (2.36)	58.67 (16.42)	66.04 (11.36)
	*arc*	23.29 (9.55)	18.92 (9.13)	3.63 (0.70)	3.04 (1.06)
Double		5.00 (2.07)	2.46 (0.87)	4.17 (1.19)	3.63 (0.96)
First cocaine (800 μg/kg) Seconds heroin (200 μg/kg)	*h1a*	18.96 (4.33)	7.57 (1.75)	50.68 (7.34)	56.46 (7.64)
	*arc*	12.11 (1.53)	12.61 (2.91)	5.17 (0.74)	4.32 (1.16)
Double		3.14 (0.59)	1.75 (0.49)	3.11 (0.46)	3.93 (0.93)

**Figure 5.**
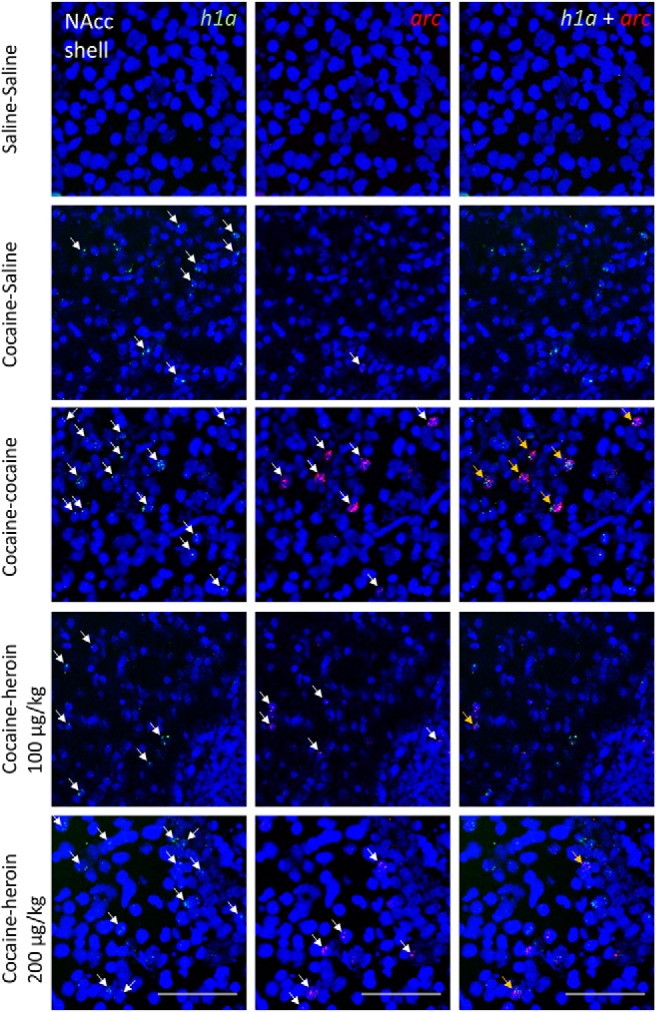
Representative microscope images taken from the NAcc shell. DAPI-stained cell nuclei (blue) coexpress only *h1a* (green), only *arc* (red), or both. The columns show green and red channels separately and then merged. Scale bars, 0.1 mm. Arrows point to mRNA^+^ nuclei.

**Figure 6.**
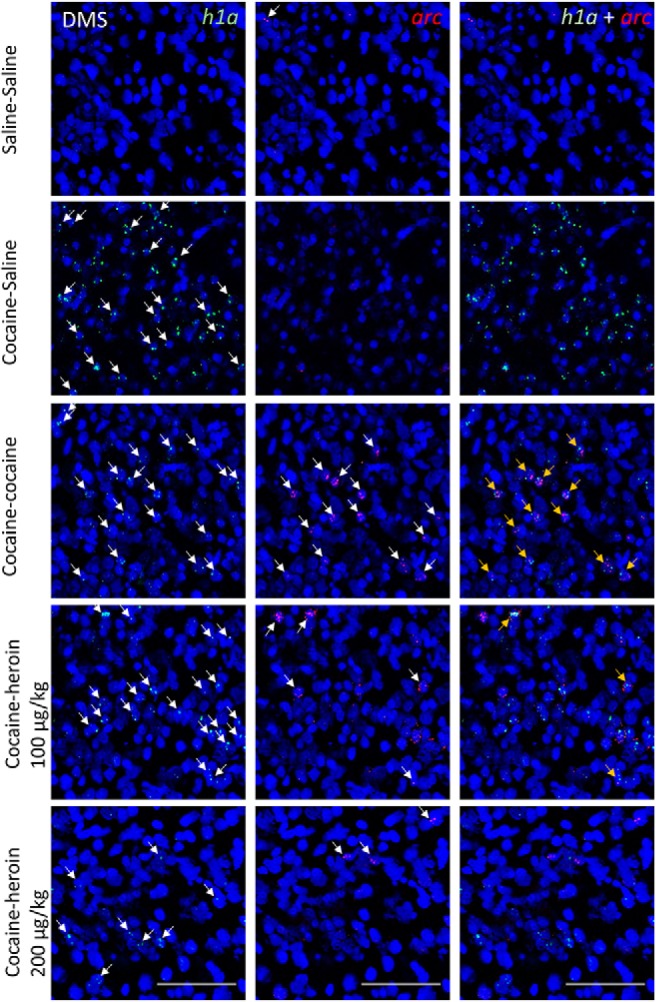
Representative microscope images taken from the DMS. DAPI-stained cell nuclei (blue) coexpress only *h1a* (green), only *arc* (red), or both. The columns show green and red channels separately and then merged. Scale bars, 0.1 mm. Arrows point to mRNA^+^ nuclei.

**Figure 7.**
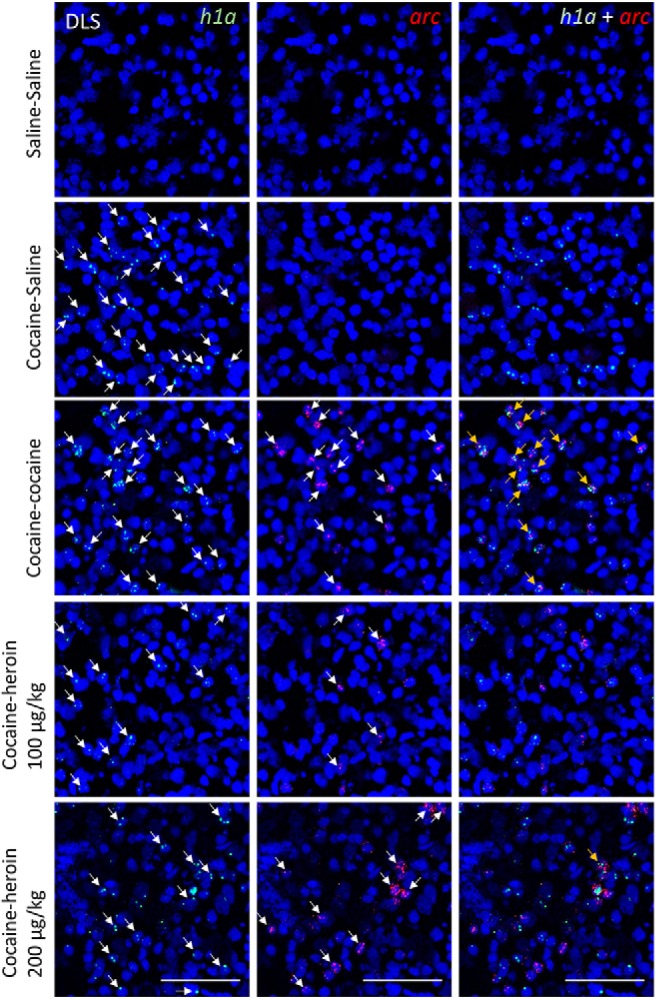
Representative microscope images taken from the DLS. DAPI-stained cell nuclei (blue) coexpress only *h1a* (green), only *arc* (red), or both. The columns show green and red channels separately and then merged. Scale bars, 0.1 mm. Arrows point to mRNA^+^ nuclei.

Figure 3*B* shows the extent of overlap between neuronal populations activated by cocaine and heroin as a percentage change from the cocaine–cocaine group. Overlap was quantified as the number of nuclei coexpressing *arc* and *h1a* expressed as a percentage of all mRNA^+^ nuclei (single and double labeled). In all four brain areas examined, there was a substantial reduction in overlap when cocaine and heroin were administered in succession, relative to the overlap seen when cocaine was administered twice, and regardless of heroin dose (Fig. 3). A two-way mixed ANOVA showed a significant main effect of treatment group (*F*_(3,19)_ = 20.97, *p* < 0.001, *η^2^* = 0.768) and brain area (*F*_(3,57)_ = 3.40, *p* = 0.024, *η^2^* = 0.152), but not treatment × brain area interaction (*F*_(9,57)_ = 0.79, *p* = 0.619, *η^2^* = 0.112).

## Discussion

We have shown that intravenous injections of heroin and cocaine at doses typically self-administered by rats produce a quick and transient increase of *homer 1a* and *arc* expression across the striatum. More importantly, using the catFISH technique, we took advantage of the difference in the timing of expression between the two IEGs to show that heroin and cocaine activate partly distinct neuronal populations in this brain area.

In line with our findings, previous studies have shown that heroin and cocaine increase c-*fos* levels in the ventral and dorsomedial striatum (Hope et al., 1992; Harlan and Garcia, 1998; Uslaner et al., 2001; Ferguson et al., 2004; Paolone et al., 2007; Celentano et al., 2009). The IEG c-*fos* is a marker of neuronal activity expressed under similar conditions of *arc* and *homer 1a* (Guzowski et al., 2001). In addition, our findings indicate that this activity occurs in separate neuronal populations and may explain why only a small proportion of neurons shows similar electrophysiological responses to heroin and cocaine (Chang et al., 1998).

It is likely that drug-induced IEG expression represents glutamatergic activity modulated by DA, because NMDA and DA D_1_ receptors play a key role in IEG expression through the activation of CREB (Impey et al., 1998; Mattson et al., 2005; Surmeier et al., 2007; Guez-Barber et al., 2011; Tritsch and Sabatini, 2012), and both DA and glutamate levels are increased in the striatum following heroin and cocaine administration. Note, however, that DA release alone does not produce IEG expression in the absence of glutamatergic activity (Kreuter et al., 2004). In addition, NMDA receptor activity and DA transmission in the accumbens are necessary for food and cocaine self-administration, but not heroin self-administration (Ettenberg et al., 1982; Pettit et al., 1984; Pulvirenti et al., 1992; Kelley et al., 1997). Finally, D_1_ receptor-expressing medium spiny neurons (MSNs) in the dorsal striatum appear to be sufficient to sustain operant behavior (Kravitz et al., 2012), and these neurons express IEGs (i.e., are activated) following cocaine administration. Thus, loss- and gain-of-function studies have provided evidence that the activity of cells in the striatum plays a key role for cocaine, but not for heroin, reinforcement through DA and glutamate transmission. The functional role of the distinct neuronal populations engaged by heroin relative to cocaine remains to be determined.

### A case for drug-specific neural circuitries

Perhaps the most intriguing interpretation of the results presented here is that partly distinct neuronal populations activated by heroin and cocaine across the striatum are suggestive of dissociated circuitry processing the acute effects of the two drugs. There is already evidence that the striatum is functionally and structurally organized to accommodate circuits that operate in parallel but carry out separate functions. First, striatal MSNs are characterized by more or less excitable states (i.e., “up” and “down” states; Wolf et al., 2001; O’Donnell, 2003), and in order for MSNs to be excited (and to express IEGs), they must receive input from several sources, which may include different combinations of amygdala, hippocampus, thalamus, PFC, and VTA/SNc afferent inputs (Pennartz et al., 1994). Each of the brain areas sending these afferent projections (1) is affected differently by heroin, cocaine, and natural rewards (Chang et al., 1998; Mukherjee et al., 2018); (2) contains neuronal ensembles involved in distinct functions (Zelikowsky et al., 2014; Warren et al., 2016); and (3) might be composed of genetically distinct projection neurons. Thus, considering the integrative function of the striatum, the diverse connectivity and specialized functions of its input regions, and the necessity for synchronized excitatory input to elicit action potentials from MSNs, it is quite possible that the activation of partly distinct neuronal populations in the striatum reflects the activation of dissociated circuitries. Here it must be noted that, although the afferent inputs of the striatum from limbic and cortical areas are topographically organized in a ventromedial–dorsolateral fashion, they are not constrained to perfectly defined striatal subregions, but are overlapping, with higher concentrations of certain afferents in, for example, shell versus core (Voorn et al., 2004). It should also be considered that MSNs send collateral GABAergic projections to neighboring MSNs. This mutual inhibition between MSNs is another functional-anatomic feature predisposing the accumbens and the rest of striatum to accommodate neuronal ensembles embedded in distinct circuitries; while one ensemble is active, it can decrease the activity in other ensembles so that only one computation is taking place over others (Pennartz et al., 1994). The experiments presented here are only suggestive of distinct striatal circuitry engaged by heroin and cocaine. Future studies should address this hypothesis by expanding on our findings in three ways. First, single-cell quantitative PCR studies can further elucidate phenotypic differences between neuronal populations activated by heroin and cocaine in terms of their genetic makeup (Hrvatin et al., 2018). Second, retrograde and anterograde labeling studies in conjunction with immunohistochemistry can reveal whether these neuronal populations connect to distinct upstream and downstream targets. And third, selective loss- and gain-of-function studies can be used to test whether inactivation of neurons responding to cocaine in the dorsal striatum and accumbens core would impair heroin reinforcement. The Daun02 technique (Koya et al., 2009, 2016) would be a useful technique in this regard, as well as other techniques that manipulate neuronal ensembles such as the TetTag approach using the Fos-tTA mouse line combined with optogenetics (Reijmers and Mayford, 2009; Liu et al., 2012; Du and Koffman, 2017).

### Methodological considerations

Two caveats to the experimental design used here are worthy of mention. There are known differences between the effects of noncontingent versus contingent exposure to heroin and cocaine (Galici et al., 2000; Lecca et al., 2007; Radley et al., 2015). In the present study, we administered heroin and cocaine in a noncontingent but unsignaled manner as we were interested in comparing the acute pharmacological effects of these two drugs using IEG expression as a marker of neuronal activation. Contingent administrations (e.g., self-administration) require repeated exposure to drugs over several test sessions, which has been shown to produce habituation to IEG expression (Hope et al., 1992; Unal et al., 2009). Of course, we recognize the value of studying the encoding of drug-related information in the striatum during and after periods of drug self-administration. Future studies could use *in vivo* imaging techniques such as the UCLA/Inscopix Miniscope to address this question directly. A second, somewhat related caveat is that our paradigm includes a multisubstance component. It is possible that circuit activity may differ following polysubstance versus single-drug use histories. However, electrophysiological evidence from rats self-administering both substances is congruent with our findings (Chang et al., 1998). Also, we administered only two injections of cocaine and/or heroin to drug-naive rats, so it is unlikely that any long-term polysubstance use effects would have influenced our observations.

### Conclusion

In summary, we found a significant dissociation in the neuronal populations responding to self-administration doses of heroin versus cocaine, as indicated by *arc* and *homer 1a* expression. Our findings provide a proof of concept that heroin and cocaine effects on the brain must be studied as separate phenomena, adding to the evidence of major differences among the various drugs of abuse (for review, see Badiani et al., 2011). Although the functional significance of these differences remains to be fully explored, they might have implications for both research and treatment. It is remarkable, for example, that the functional or anatomic integrity of the dopaminergic system is required for the reinforcing properties of cocaine but not of heroin (Ettenberg et al., 1982; Pettit et al., 1984; Pisanu et al., 2015), that distinct projections from the PFC to the shell of the NAcc are implicated in the relapse to cocaine versus heroin seeking after abstinence (Peters et al., 2008; Bossert et al., 2012), and that basic environmental manipulations gate in opposite directions the reinforcing, affective, and neurobiological responses to heroin versus cocaine in rats and humans (Uslaner et al., 2001; Ferguson et al., 2004; Caprioli et al., 2007a, 2008, 2009; Paolone et al., 2007; Celentano et al., 2009; Montanari et al., 2015; Avvisati et al., 2016; De Pirro et al., 2018; De Luca et al., 2019).

## Synthesis

Reviewing Editor: Yavin Shaham, NIDA-IRP/NIH Decisions are customarily a result of the Reviewing Editor and the peer reviewers coming together and discussing their recommendations until a consensus is reached. When revisions are invited, a fact-based synthesis statement explaining their decision and outlining what is needed to prepare a revision will be listed below. The following reviewer(s) agreed to reveal their identity: Susan Ferguson.

The reviewers found merit in the study but differed in their overall evaluation. Based on my reading of the paper, I do not think that additional experimental data are necessary for a successful revision, but it is important to fully address the critical comments of reviewer 1 regarding experimental design and interpretation of the data. I enclose below the individual comments of the reviewers and my recommendation of how to address them in the revision. In addition to their comments, also address the comments below:

1. Abstract: please use the 250 words allocated to this section to provide additional experimental details (drug doses, route of administration, location of injections, striatal regions, etc) and additional description of the empirical results. Also delete citations from this section

2. Introduction: use first author et al. format for text references

3. Methods and other places in the text: please replace 'animals' with 'rats' when referring to the experimental subjects

4. Discussion: please add 2-3 informative sub-headings to orient the readers of the topics being discussed

5. References: please use eNeuro/J Neurosci formatting

6. Figures 4-7: please label in the figure the brain area

7. Figure legends: please add n per group

REVIEWER 1

The authors have presented a study that uses the catFISH technique, based on timing-dependent activation of two immediate early genes, to investigate differences in neuronal activation in the striatum following noncontingent cocaine and heroin administration. While the study overall is logical and of interest, there are significant methodological concerns and missing rationale that limit the potential impact of the work and need to be addressed.

Major concerns:

1. The significance statement refers to a “widely accepted idea of a common neurobiological substrate for all drug addictions,” but the introduction is filled with references that contradict that statement. The wording should be substantially damped down to more accurately describe the state of the field.

Editor: please revise this section to address the reviewer's concern

2. In the introduction, please provide more discussion on the selection of homer 1a and arc in this study (e.g., whether they employ similar signaling cascades, whether stimuli cause equal activation of the two). Additionally, please explicitly state the underlying hypothesis and rationale - as it is currently written, this study appears more as a fishing expedition than a study with a grounded hypothesis.

Editor: please revise this section to address the reviewer's concern

3. Significant methodological details are missing, including how catheter patency was maintained, how and when catheter patency was confirmed, the source of cocaine and heroin, how cocaine and heroin were prepared for iv infusion (concentration, volume, rate), and what alpha value was used for determining significant effects. Additionally, description of the timecourse for Experiment 1 is very odd - were rats euthanized at timepoints relative to pentobarbital injection (as written), or relative to the cocaine/heroin injections?

Editor: please add the missing experimental details

4. Why was an 18h habituation period used for Experiment 2? Water and food restriction are likely to have a confounding impact on interpretation of the data. Moreover, it is unclear why self-administration chambers were used at all, since the infusions were given non-contingently. This needs to be specifically addressed.

Editor: please provide a rationale for the different contexts in Experiments 1 and 2 and address the reviewers' comments on water and food restriction

5. Why was a heroin/heroin or heroin/cocaine group not included in this study? Why wasn't the injection order counterbalanced for each group? Dose responses are needed to ensure that the different patterns of induction are not due to dose. In addition, the overall cell counts for each group are quite low, which is likely due to unsignalled infusions in home cages or following long habituations. Additional experiments using signaled infusions/novel environments and/or following self-administration would strengthen the study greatly.

Editor: as mentioned above, additional experiments are of course welcome but not mandatory for a successful revision. At a minimum, please provide a rationale for not counterbalancing the heroin/cocaine order and for not running the heroin-heroin condition

6. The reporting in the results section is highly redundant and difficult to follow. More importantly, however, is the choice of the authors to simply list their ANOVA summaries without including any post-hoc comparisons. While problematic across-the-board, this is particularly concerning for the NAc core h1a expression data for heroin, in which there were no significant main effects or interactions. If heroin is not inducing h1a expression and cocaine is, this is a serious confound for Experiment 2. How do the authors rationalize this? Analyzes should be represented on figures with symbols.

Editor: please address this comment of the reviewer. I agree with the reviewer that the Result section can be shorter and this can be achieved by deleting means/sem that are depicted in the figures from this section

7. The data for experiment 2 are listed as percent change from cocaine/cocaine group, which is problematic and misleading, particularly given that there is not l large degree of co-localization between genes in that group and that a saline/saline group was run. As heroin was already shown to not activate h1a in the NAc core (Experiment 1), it seems deceptive to show data relative to cocaine, which does activate h1a. Data should be shown as total number of positive nuclei. If percent changes are shown, these should be from the proper control group (saline/saline).

Editor: Based on the reviewer's comment, I suggest showing the actual number of individual and double-label neurons instead of percent values

8. A rationale for and larger discussion of the caveats of examining cocaine and heroin use in a noncontigent model is necessary, given that the activation of cortico-basal-ganglia circuitry may be significantly impacted by the absence of goal-directed behaviors, and how contingent administration may result in differences in overlap in this activity.

Editor: please revise the text to address the reviewer's concern. I recommend adding a section on Methodological and interpretation considerations to the revised Discussion

9. As the paradigm implemented a multi-substance component, discussion should be included on the implications of circuitry activation following polysubstance use and how it may compare to single drug histories.

Editor: please add text to address the reviewer's comment

Minor concerns:

1. Figure 4 includes arrows pointing to mRNA-positive nuclei, but the other similar figures (5-7) do not have this. Arrows should be added to these other figures to assist comparison across brain regions.

2. “D1” is used as an abbreviation for both “drug 1” (Figure 1 legend) and the dopamine D1 receptor (Discussion).

3. Typo, line 114: “sections were cut at on a cryostat”

4. Typo, line 150: “analyzsed using the RIO Montepellier extension”

Editor (minor comments 1-4): please revise the figures and text to address the reviewer's comments

REVIEWER 2

Using the catFISH technique, the authors demonstrate that intravenous injections of cocaine followed by heroin activate non-overlapping neurons in the ventral and dorsal striatum. The introduction and discussion are very well-written and the methods are sound. This is an important proof-of-concept paper that will be well-received by the drug addiction research community. I have only a few comments:

1. Please include figure titles

Editor: please add the titles

2. I cannot see the labeling of arc in the red channel very well, either on my computer screen or the print copy.

Editor: please improve the quality of the figures

3. Labeling arrows should be included in figures 5-7 (like they are in figure 4)

Editor: please add the arrows

4. I am not sure if it is correct to say “activate largely distinct neuronal populations in this brain area” because the overlap was 45-50% in accumbens shell and core which is about 1/2 overlap

Editor: Based on the reviewer's comment, I suggest using the term 'partly overlap' to describe the data

5. Line 68: “This was done to avoid injury from fighting once cannulas were implanted.” Researchers generally single house rats after surgery to prevent them from chewing on the others' cannula, not because of fighting (and rats play, not fight, with their housing partner)

Editor: please revise the text based on the reviewer's comments

6. Line 87: missing “after a” between “rats” and “few seconds”

7. Line 323: change “into” to “to”

Editor: please correct the text
